# Dioxin Exposure in the Manufacture of Pesticide Production as a Risk Factor for Death from Prostate Cancer: A Meta-analysis

**Published:** 2018-02

**Authors:** Ali KABIR, Rezvan ZENDEHDEL, Raana TAYEFEH-RAHIMIAN

**Affiliations:** 1. Minimally Invasive Surgery Research Center, Iran University of Medical Sciences, Tehran, Iran; 2. Dept. of Epidemiology, School of Health, Shahid Beheshti University of Medical Sciences, Tehran, Iran; 3. Dept. of Occupational Health Engineering, School of Health, Shahid Beheshti University of Medical Sciences, Tehran, Iran

**Keywords:** Dioxin, Meta-analysis, Pesticides, Prostatic neoplasm

## Abstract

**Background:**

In pesticide exposure groups such as farmers, the risk of prostate cancer was increased, although the report of the cause of evidence is limited. We selected chlorophenol compounds as an important group of the contaminated pesticide with highly toxic 2, 3, 7, 8-tetrachlorodibenzo-p-dioxin (TCDD). This meta-analysis, the risk of death from prostate cancer was analyzed.

**Methods:**

PubMed, Scopus, Scholar Google and web of Sciences until 2016 were searched. The standardized mortality rate (SMR) and 95% confidence intervals (CI) were obtained from the studies. We tested statistical heterogeneity with Cochrane Q test and I2 index. Egger test was used for evaluating publication bias. Random or fixed-effects models and meta-regression were also used in our analysis. Moreover, Cochrane tool was used to assess the risk of bias.

**Results:**

Five available papers consist of 28706 exposed populations were assessed. Overall standardized mortality rate as combined result of prostate cancer risk from the fixed model was 1.2 (95% confidence interval (CI) 1.02 to 1.42, *P*=0.027). Some biases are more probable in these studies such as confounding by indication, loss to follow up and misclassification.

**Conclusion:**

A contaminated pesticide with dioxins between other pesticides is an important risk factor for prostate cancers.

## Introduction

Prostate cancer is the most common cancer in men the worldwide (1, 2). Age and genetic basis are well-established etiologies for prostate cancer (3). In addition, there is considerable evidence for environmental and occupational factors for inducing prostate cancer (4, 5). Adverse effects of pesticide exposure to reproductive functions have been reported in laboratory animal studies (6, 7). Moreover, pesticide exposures may cause endocrine disruption and the excess risk of prostate cancer in human (8). The use of large amounts of pesticide imposes unwanted contaminated pollutant as destructive health of humans (9). Chlorophenols (CPs) and phenoxy acetic acids (PAs) are groups of pesticides used for the control of insects and weed. Some phenoxy herbicide production based on CPs (10) was contaminated to 2, 3, 7, 8-tetrachlorodibenzo paradioxin (TCDD). Because of TCDD dependency, CPs has been classified as a limited pesticide by the Environmental Protection Agency (11). TCDD is the most toxic congener of dioxin (12). Since IARC evaluation for TCDD carcinogenicity classified in Group 1 as a known human carcinogen (13), also some reports confirm endocrine disruptor effects of them (14, 15).

Much of the research demonstrate mortality of soft-tissue sarcoma and non-Hodgkin's lymphoma in chlorophenol exposure contaminated to TCDD (10, 16), whereas less report has been paid for endocrine toxicity. Some studies have reported a positive association between TCDD exposure and prostate cancer (17, 18), while others have not (19, 20).

In this study, we conducted a meta-analysis of the literature focused on the association between dioxin exposure among workers in manufacture of pesticides producing and prostate cancers.

## Methods

### Literature search and inclusion criteria

We collected all studies of prostate cancer in chlorophenol compounds production through 2016 by using PubMed, Scopus, Scholar Google (until first 20 pages), and Web of Sciences databases using different language. The key terms for searching were “pesticide”, “chlorophenol”, “phenoxy acetic acid”, “prostate”, “cancer”, “neoplasm” and “dioxin” with different combinations. The references of articles were manually searched to find related publications.

Overall, 784 studies were included. After checking titles and abstracts of all publications 25 studies were selected which reported cancer in CPs production factory. After reviewing the full text of these papers, 13 studies were selected based on the following inclusion criteria:

- Publications that report the risk of prostate cancer
- Publications including prostate cancer risk in workers of production factory with chronic exposure to CPs.
- Prostate cancer risk reporting in accidental exposure was excluded.

By comparing the full text of these 13 studies, six publications (21–26) were identified which had overlap with one international cohort study (27). We included this international study (27) as the replacement of overlapping studies. Two other papers (17, 18) overlapped with other publications (20, 29).

**Fig. 1:**
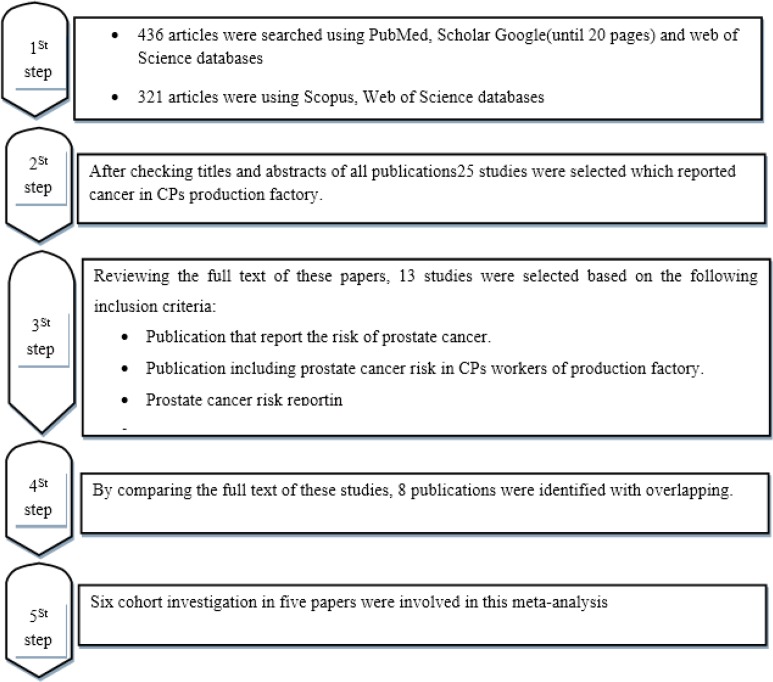
Flow diagram of publication inclusion in the meta-analysis

Therefore, five published articles (19, 20, (27–29) were included in the meta-analysis. There was a paper that has two independent cohort investigations with different entry population (20). Finally, six-cohort investigations in five papers were involved in this meta-analysis (Fig. 1).

### Data extraction

Response rate and 95% confidence interval, the country where study was accomplished, period of study, year of publication, study design (cross-sectional, case-control, or cohort), sample of exposed group, co-morbidities, time of exposure, number of authors and journal impact factor (IF) in the year of publication was extracted from each study. In this study, response rate of exposed group was classified by latency of effect and type of component exposure. We asked authors some other information, which there was not in the papers. They E-mailed some of our questions except for place of the study (district, and rural/urban area).

### Study quality and risk of bias

The quality of study was assessed by STROBE guideline [www.strobe-statement.org available at 8 April 2013]. In order to assess the risk of bias (i.e., internal validity), Cochrane tool was used. We evaluated selection bias (e.g. non-response, survival, and healthy worker), information bias (e.g. misclassification, surveillance), confounding bias (e.g. susceptibility, confounding by indication, migration or exchange) and loss of follow up for each study. Overall assessment risk of bias for each study classified as green (low risk of bias), yellow (unclear risk of bias) or red (high risk of bias) according to each key criteria.

### Data analysis

Standardized mortality ratios and 95% CI were evaluated for each study. To test comparability among studies; we tested the homogeneity between the studies with Cochrane Q test. The degree of heterogeneity was estimated using I^2^ factor where I^2^ < 25% have low, between 25% to 50% moderate, and I^2^ >50% high degree of heterogeneity (34). If heterogeneity was not observed between the studies (Q-test *P*-value was ≥0.1) a fixed-effects model of Mantel-Haenszel test was used to calculate the meta SMRs. Finally, the random effects model based on DerSimonian and Laird method was evaluated when studies were heterogeneous.

Validity of meta-analysis was assessed by publication bias based on Egger test and Begg’S funnel plot. Meta-regression used for exploring the significant sources of heterogeneity while we considered all probable variables that could change tau index. Begg’s funnel plot was characterized by plotting the logarithm of prostate cancer SMR versus the standard error where an asymmetry around Begg’s funnel plot specifies sources of heterogeneity.

## Results

Five papers with six cohort reports published data on the association of dioxin exposure in CPs production and prostate cancer that total of 28706 exposed populations involved in the analysis. Fifty-two percent of populations included in the studies were from the USA and 48% from Europe. The study (18, 20) appraised exposure with different types of CPs.

**Fig. 2:**
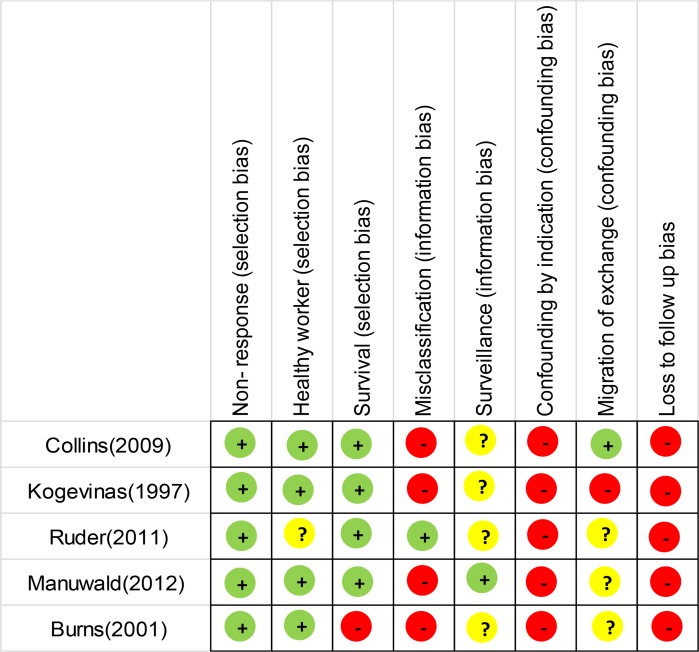
Risk of bias in all five studies included in meta-analysis

A publication of IARC study covering 36 previous studies from 12 countries when it followed prostate cancer in pesticide production from1939 to 1992 (27). Loss to follow up, confounding bias and misclassification bias was the most common biases that these five studies were involved with them. Other biases were less probable (Fig. 2).

**Fig. 3:**
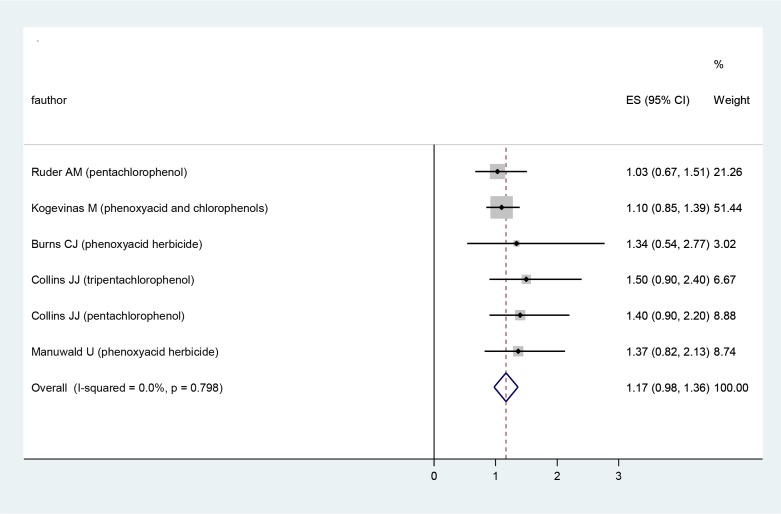
Forest plot of SMR and their 95% CIs from dioxin exposure and prostate cancer risk according to fixed effects model

No Significant heterogeneity existed among the combination of six SMRs reported in the studies (*P*-value=0.93). The degree of heterogeneity was estimated low based on the I^2^ <25%. The fixed effect model was applied in this analysis and produced a meta-rate ratio of 1.2 (95% CI 1.02 to 1.42, *P*=0.027) where yielded 20% additional risk for death from prostate cancer in exposed group (Fig. 3).

Once we excluded the study (27) which followed 21863 exposed persons and has 43.93% of total weight of our meta-analysis, we obtained a summary SMR of 1.29 (95% CI 1.037–1.61; *P*=0.03). Meta-analysis excluding the study (27) revealed no major difference in risk of prostate cancer-related death in comparison with combination of all other studies. Information on the type of dioxin-contaminated pesticide exposure was reported for three groups of component. Two studies reported data on CPs exposure only (18, 20), three publications on PAs exposure (17, 28, 29) and two others reported for the mixture of CPs and PAs exposure (19, 28). CPs exposure was associated with prostate cancer risk significantly (Meta: SMR = 1.32, 95% CI = 0.989–1.751, Q-test *P*-value = 0.742).

We did not demonstrate any significant relationship between each one of factors like period of study, year of publication, study design, sample of exposed group, time of exposure, number of authors, journal impact factor (IF) and latency of exposure which sample had exposure with them and SMR of prostate cancer. However, when we sorted studies based on their midpoint year of running the study, there was an increasing trend for SMR of prostate cancer, except the newest study [28], which had a decreasing index in comparison with its previous study (Fig. 3).

## Discussion

In recent years, the incidence of the risk of prostate cancer mentioned as one of the most frequently reporting (1, 2). Occupational factors play an important role in the evidence of this disorder (4). These meta-analysis results indicate association between dioxin exposure and increased risk of prostate cancer, a finding that did not major change whether or not we included the study (27) as a study with highest weight in meta-ratio risk.

By bias assessment, only one report can be reliable among included studies in meta-analysis. Different types of selection bias were less probable. Therefore, the results of these studies are much probably generalizable to other cases with prostate cancer at risk of exposure to dioxin compounds. However, internal validity of these studies due to different types of information bias is under question. Sensitivity analysis in these studies could be a solution for compensating for loss to follow up.

In the previous meta-analysis, the association between pesticide exposure in different occupation (30) and prostate cancer (31) was reported; however, type of component exposure or the class of position was not illustrated where significant heterogeneity existed among these studies. In our meta-analysis, appropriate exposure to dioxins in chlorophenols production indicates no heterogeneity and significant increase in the risk of prostate cancer. However, any evidence did show a link between amount of serum TCDD with testosterone levels and benign prostatic hyperplasia (32).

Our analysis supports a significant of 20% increase in SMR of prostate cancer for population with dioxin exposure. Early historical cohort studies by the International Agency for Research on Cancer were covering 36 previous studies in the USA and Europe from 1939 to 1992 (27). Rate ratios in this study (SMR: 1.1, 95% CI 0.85 to 1.39) were higher than 1 alike meta-rate ratio of our study; but, there were not significant. Combining studies published after 1997 (19, 20, 28, 29) gave a significant rate ratio (SMR: 1.29) of prostate cancer. Moreover, the newest publication (28) showed a decreasing SMR of prostate cancer in CPs producing. This may be caused by limitation of CPs and PAs usage, control measures in occupational health and different process in pesticide production.

In different exposure of chlorophenols and phenoxy acetic acids, CPs exposures significantly highlighted development of prostate cancer when a non-significant increase of prostate cancer was reported in workers exposed to CPs pesticide (18, 20).

One reason for absence of significant relationship between each one of factors like period of study, year of publication, study design, sample of exposed group, time of exposure, number of authors, journal impact factor (IF) and latency of exposure and SMR of prostate cancer could be related to low number of investigated studies in our meta-analysis.

However, additional unknown factors might also be applied since the lack of smoking and dietary habits, and intake of alcohol information on the incidence of prostate cancer may play a role and we had no information about them in these studies as a limitation. Extensive in vitro and in vivo toxicity testing of dioxin-contaminated chlorophenols has shown activity of androgen receptor modulators effect for these compounds which stimulates cell proliferation (33, 34). Finally, the results of our meta-analysis confirmed toxicological findings in the prostate cancer incidence and dioxins exposure.

## Conclusion

Our findings has strengthened the evidence of prostate cancer in occupational exposure to chlorophenols. These results confirmed increase of death risk from prostate cancer.

## Ethical considerations

Ethical issues (Including plagiarism, informed consent, misconduct, data fabrication and/or falsification, double publication and/or submission, redundancy, etc.) have been completely observed by the authors.
